# Real-Time Efficient Relocation Algorithm Based on Depth Map for Small-Range Textureless 3D Scanning

**DOI:** 10.3390/s19183855

**Published:** 2019-09-06

**Authors:** Fengbo Zhu, Shunyi Zheng, Xiaonan Wang, Yuan He, Li Gui, Liangxiong Gong

**Affiliations:** 1 School of Remote Sensing and Information Engineering, Wuhan University, Wuhan 430079, China; 2 Wuhan Zhongguan Automation Technology Co., Ltd., Wuhan 430066, China; 3 Collaborative Innovation Center of Geospatial Technology, Wuhan University, Wuhan 430079, China; 4 Nanchang Institute of Surveying and Mapping, Nanchang 330013, China

**Keywords:** real-time relocation, global relocation, key point detector, feature matching verification, transformation estimation

## Abstract

As an important part of industrial 3D scanning, a relocation algorithm is used to restore the position and the pose of a 3D scanner or to perform closed-loop detection. The real time and the relocation correct ratio are prominent and difficult points in 3D scanning relocation research. By utilizing the depth map information captured by a binocular vision 3D scanner, we developed an efficient and real-time relocation algorithm to estimate the current position and pose of the sensor real-time and high-correct-rate relocation algorithm for small-range 3D texture less scanning. This algorithm mainly involves feature calculation, feature database construction and query, feature matching verification, and rigid transformation calculation; through the four parts, the initial position and pose of the sensors in the global coordinate system is obtained. In the experiments, the efficiency and the correct-rate of the proposed relocation algorithm were elaborately verified by offline and online experiments on four objects of different sizes, and a smooth and a rough surface. With more data frames and feature points, the relocation could be maintained real time within 200 ms, and a high correct rate of more than 90% could be realized. The experimental results showed that the proposed algorithm could achieve a real-time and high-correct-ratio relocation.

## 1. Introduction

Recently, some high-precision three-dimensional (3D) handheld scanners, such as GoScan [1], EinScan-Pro+ [2], and GSCAN [3] have been used in 3D modeling, industrial inspection, and reverse design. These 3D scanners are based on the binocular stereo visual technology, which captures the two images with structured light specular once and generates the depth map by using a stereo matching method. These devices can generate a more accurate 3D depth map because of the smaller scanning distance. However, because of the small scanning distance, the range of the depth map is small. When the scanner moves too fast, or the scanner moves directly to another location to scan the rest of the region, this small-range scanner unavoidably loses its position and pose. Restoring the pose and the position of the scanner requires the ability of relocation. The information that we can use is only the images captured by the cameras and the depth map information obtained through stereo matching. However, in the case of industrial mold scanning or gypsum sculpture scanning, the texture of the scanning object is often scarce; in other words, it is difficult to locate the scanner by using texture information. Therefore, the depth map is used to locate the scanner.

The process of scanning can be divided into tracking and mapping. In the tracking part, the current depth map is used to register with the previous frame’s depth map or the depth map generated by the raycasting method [4]. The registration result is a 6-degree of freedom (DOF) transformation matrix. In the mapping part, the global frame data are generated by transforming the current depth map by using the transformation matrix; these global frame data are then fused into the scanning model by using the truncated signed distance function (TSDF) method [4]. When the tracking is lost, the current depth map is used to relocate the initial position and pose of the scanner; then, the refined position and pose can be obtained by registering the current depth map to the model map generated by using the initial position and pose. We focused on the recovery of the global pose and position of the scanner by using a depth map when tracking failed.

Depth maps have been used for 3D object recognition [5], 3D registration [6], 3D modeling [7], 3D object search [8], etc. However, no related studies can be found on small-range sensor relocation using depth maps in the incremental scanning process. In 3D object recognition [9], an intrinsic shape signatures (ISS) feature description is proposed and a query database is built for searching similar objects, which achieves a high recognition performance; however, the focus is on finding a model similar to the current radar scan model from the computer aided design (CAD) model library, instead of getting the position and the pose. 3D pairwise registration often uses the iterative closest point (ICP) [10] or its variants [11,12,13,14] for registration to obtain the transformation relationship; however, the ICP or its variants are sensitive to the initial values. Some better rough registration methods based on features have been proposed to solve the initial value problem [15,16,17]; these algorithm pipelines can generally be summarized by the following three steps: (1) Point cloud features such as globally aligned spatial distribution method (GASD) [18], ISS, and signature of histograms of orientations (Shot) [19] are extracted from the point clouds. (2) The initial matching features are obtained, which meet the similar distance measure of the feature descriptor. (3) Some matching verification methods, such as Hough vote [15], geometric consistency (GC) [20], and search of inliers (SI) [21], are used to filter out the wrong matching features and obtain the registration matrix. Although these feature-based coarse registration methods solve the initial value problems, they are not suitable for incremental 3D reconstruction, because as the amount of scan data increases, the model becomes larger, and thus extracting the feature points and finding the initial matching points on the model will be a very time-consuming process.

Features are successfully applied in the coarse registration method. They are divided into global features and local features [22]. Global features are not suitable for point cloud pair registration, which only has a partially overlapping region [22]. The local features describe the spatial or geometric properties of the neighborhood of the feature point. Compared with the global features, the local features are more suitable for pairwise alignment [22]. Therefore, these features were used to recover the position and the pose of the sensors in this study. Then, we were devoted to solve the following four problems: (1) How to extract the feature points from the current frame depth map effectively? (2) How to construct the feature query database and query the features quickly? (3) How to filter out the wrong matching feature point pair effectively? (4) How to get a correct transformation matrix?

A few local feature extreme points that represent the geometric characteristics of the whole data are extracted, which can reduce the information redundancy. Thus far, 3D feature extraction has been extensively studied, and many different 3D feature descriptors [23,24,25,26,27] have been proposed. The local reference frame (LRF) is an independent coordinate system constructed on the local 3D surface. It is broadly used in 3D local feature descriptors, and the LRF can make the local feature descriptor with rotational invariance. Many different LRF calculation methods [28,29,30,31,32,33] have also been proposed to get a rotation-invariant local feature descriptor. The stability of the extracted feature points is an important problem. The eigenvalues ratio and the single eigenvalue constraint are used to improve the stability of the feature points [9,34,35]. In the actual scene scan, the depth map of each frame contains some invalid data because of occlusion, deep slot, and sharp protuberance. The eigenvalues constraint and the eigenvalues ratio constraint usually lead to the location of the feature points on the edge of the invalid data. The scanning of different perspectives leads to different invalid data edges, which will result in unstable features. These unstable features affect the feature matching and transformation calculations.

An efficient similar feature search strategy is also an important issue in the incremental reconstruction process. An appropriate feature organization structure can accelerate the similar feature queries and insertions. The easiest way to do so is to perform a brute-force search to query all the similar features, but as the number of feature points increases, the search time will increase rapidly. The bags of binary words (DBoW2) [36] method is commonly used in relocation and closure detection in simultaneous localization and mapping (SLAM). However, as an offline training method, it cannot be applied to different unknown scenes. KD-tree [37] is a fast and accurate method to get the nearest feature; however, KD-tree needs to be rebuilt every time when new feature points are added to the tree, which costs time. The local sensitive hash (LSH) [38,39,40] method is a method of approximate nearest neighbor (ANN), which is mainly applied for offline image retrieval. A local sensitive hash tree (LST) [9] is used to search for similar objects; although this method is an ANN method, its structure is very beneficial for feature query and insertion.

In the scanning process, similar features may be distributed in different spatial locations; this makes it difficult to query the correct matching of the feature. Some matching verification methods should be used to get the correct matching feature from a large number of similar features. Some methods, such as the nearest neighbor (NN) and the nearest neighbor similarity ratio (NNSR) [41], use the feature description distance measure to get the matching feature, but they ignore the geometric consistency. The GC algorithm uses feature distance verification to get the similar features and uses clustering methods to verify the geometric consistency. A 3D Hough vote [15] is a good method to verify the geometric consistency; however, it needs to allocate a large amount of memory when the target object is large. The random sampling method [42] can obtain the model fitting result with the greatest confidence, but when there are a large number of mismatched point pairs, it may get wrong results. The SI method uses a feature descriptor distance ratio limitation, point pair distance constraints, and a global voting method of point-to-point transformation to construct a global vote, and the adaptive thresholding method is used to obtain the inner points. The consistency ranking vote method [43] ranks the scores of the similar features, and the geometric consistency measure is used to get the correct matching features. We proposed a new verification method: first, we used the NN method to get the similar features, and then, the mismatched features were filtered out by the distance, angle, and direction verification. Finally, the cluster method was used to get the clustered group with a high confidence.

The 6-DOF transform optimization is a mature method. When the matching feature point pairs with high confidence are obtained, the optimized transformation matrix can be quickly obtained by the ICP or its variant methods combined with the RANSAC method. However, when there are duplicate structures in the scene, the random sampling method produces different and reliable results, which may cause a relocation failure.

We developed a real-time and efficient relocation pipeline. In the feature calculation part, more constraints were used to avoid the feature points from falling in an unstable place. Then, LST was used to perform the similar feature query and insertion. After the similar features were queried by LST, a distance, angle, and direction verification method was used to filter out some mismatching feature point pairs, and the cluster method was used to get the clustered group with a high confidence. Finally, we used the improved RANSAC sample method to avoid the interference of the duplicate structures. Therefore, this paper makes the following contributions:

- This paper presents a real-time and high-correct-rate algorithm pipeline for relocation by using depth maps in the incremental reconstruction scanning process. We divided the relocation algorithm into four parts for the first time: feature calculation, feature database construction and query, corresponding feature verification, and rigid transformation calculation.
- In the feature calculation step, we used the normal to get the LRF, and the feature descriptor was normalized to reduce the effect of the different resolutions and scanning distances. The area limitation was used to get a more stable feature point. In the feature matching verification part, we added an eigenvalue limitation to quickly get a coarse matching feature. The distance, angle, and direction verification method and the dynamic cluster method were used to filter the error matching feature pairs. In the rigid transformation calculation part, we improved the RANSAC method to avoid the interference of similar structures.

The remainder of this paper is organized as follows: the details of the relocation pipeline are presented in Section 2. In Section 3, we provide relocation experiments and discussions. Finally, we draw conclusions and outline some ideas for future work in Section 4.

## 2. Methods

After reviewing the related work on feature calculation, feature organization and query, feature matching verification, and transformation calculation, we focused our work on the real-time and high-correct-ratio global relocation algorithm.

**Figure 1:**
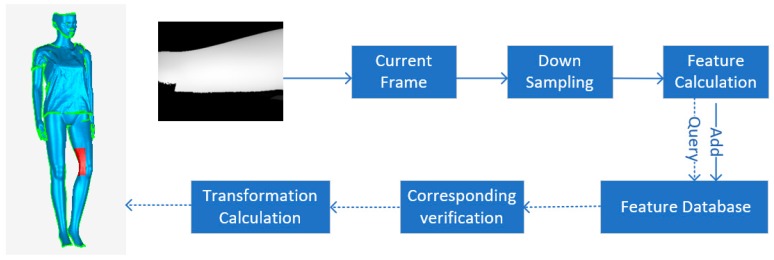
Proposed global relocation algorithm pipeline.

The pipeline of the proposed relocation algorithm is shown in Figure 1. As shown, the current depth map captured from the scanner was downsampled to accelerate the computation. Then, features were extracted from the downsampling depth map. When the current frame tracking succeeded, the features were added directly to the feature database. When the current frame tracking failed, the current frame features were used to query similar features from the feature database, the match verification part filtered some incorrect matches, and the rest of the corresponding feature point pairs were used to calculate the transformation matrix that transformed the current frame to the global model coarsely. The transformation matrix could be viewed as the initial global position and pose of the scanner. The ordered details on the relocation algorithm of the proposed pipeline are as follows.

### 2.1. Downsampling

The downsampling method was used to reduce the data and accelerate the calculation, which can be expressed as follows:

(1) $$\{\begin{array}{c} w^{\prime }=\;w/s\\ h^{\prime }=h/s\\ focus^{\prime }=focus/s\\ x_{0}{}^{\prime }=\;x_{0}/s\\ y_{0}{}^{\prime }=\;y_{0}/s\\ depthM^{\prime }=submap\left(depthM\right) \end{array}$$

where s is the sample rate; submap() function represents that the depth map is downsampled at equal intervals of s. Further, w, h, focus, $\left(x_{0}{}_{},y_{0}\right)$, and depthM represent the width, the height, the focus, the image principal point, and the depth map data, respectively. Next, $w^{\prime }$, $h^{\prime }$, $focus^{\prime }$, $\left(x_{0}^{\prime },y_{0}^{\prime }\right)$, and $depthM^{\prime }$ represent the width, height, focus, image principal point, and depth map data, respectively, after the depth map downsampling. Through experimental observations, we found that the setting of s = 4 still maintained a satisfactory performance.

### 2.2. Feature Calculation

An LRF is a 3D coordinate system established on the local surface. It is important because feature descriptions based on the LRF are invariant to a rigid transformation [44]. The neighboring points of point p were used to calculate LRF by the covariance decomposition (CA). In this paper, the LRF was calculated on the basis of the covariance decomposition of the normals of the neighbor points. The advantage was obvious: the sum of three eigenvalues of CA was 1, which reduced the difficulty of setting the thresholds. The method of CA was used to calculate the normal direction of each point on the depth map as follows:

(2) $$Cov\left(p\right)=\frac{1}{N\left(p\right)}\underset{q\in N\left(p\right)}{\sum }\left(q-\bar{q}\right){\left(q-\bar{q}\right)}^{T}$$

where q is the point in the neighborhood of point p, N(p) is the number of neighboring points, and $\bar{q}$ is the average position of the neighboring points. The eigenvectors and eigenvalues could be calculated from CA of Cov(p); the normal $n_{q}$ is the eigenvector corresponding to the minimum eigenvalue. Moreover, the normal keeps the direction towards the viewpoint. Then, the normals of the neighboring points were used to obtain LRF as follows:

(3) $$Cov\left(n\right)=\frac{1}{N\left(p\right)}\underset{q\in N\left(p\right)}{\sum }n_{q}n_{q}{}^{T}$$

The eigenvalues $\lambda_{1},\lambda_{2},\;\mathrm{and}\;\lambda_{3}\;(\lambda_{1}>\lambda_{2}>\lambda_{3})$ were obtained from the CA of Cov(n), the eigenvector $lrf_{x}=\left\{x^{\prime },y^{\prime },z^{\prime }\right\}$ corresponding to the eigenvalue $\lambda_{3}$ was taken as the local *x*-axis direction of the feature, and the feature vector $lrf_{y}=\left\{x^{\prime \prime },y^{\prime \prime },z^{\prime \prime }\right\}$ corresponding to the feature value $\lambda_{2}$. was used as the feature’s local *y*-axis direction. The eigenvector $lrf_{z}=\left\{x^{\prime \prime \prime },y^{\prime \prime \prime },z^{\prime \prime \prime }\right\}$ corresponding to the eigenvalue $\lambda_{1}$ was taken as the local *z*-axis direction of the feature.

#### 2.2.1. LRF Disambiguation

Firstly, we removed the ambiguity of the *z*-axis of the LRF. The direction of *z*-axis could be corrected by the position of the viewpoint. In each depth map, the center point of the cameras was viewed as the viewpoint, in the coordinate system of the camera. When the position of the camera center was (0, 0, 0), the direction of the *z*-axis was determined as follows:

(4) $$lrf_{z}=\{\begin{array}{l} \left\{x^{\prime \prime \prime },y^{\prime \prime \prime },z^{\prime \prime \prime }\right\}\;\;\;z>0\\ \left\{-x^{\prime \prime \prime },-y^{\prime \prime \prime },-z^{\prime \prime \prime }\right\}\;z<0 \end{array}$$

Secondly, we removed the ambiguity of the *x*-axis of the LRF. We obtained the *x*-axis direction by using this method described in the triple orthogonal local depth images (TOLDI) [33]. Then, the *y*-axis direction was determined by using the cross-product of *z*-axis and *x*-axis.

#### 2.2.2. Feature Extreme Point Extraction

Compared with the feature extreme point selection method based on the neighborhood [15], the grid-based selection method was faster. The current frame was divided into N × M grids, and a feature extreme point was expected to be selected in each grid. The feature extreme points had to satisfy the following conditions:

1. The ratio of the number of neighborhood points to the size of the neighborhood window should meet the threshold. Assuming that the neighborhood radius is r and the depth of the current point is z, the r/focus × z depth represents the radius of the pixel window. Further, assuming that the width and the height of the window are w and h and the number of points in the radius r is num, the ratio should satisfy $num/\left(W\times H\right)>\tau_{area}$.
2. In each grid, the feature points with the maximum $\lambda_{3}$ are selected as the local feature extreme points. In order to avoid the interference of the feature extreme points falling on the edge of the grid, the feature extreme point should be verified in its eight neighborhoods.
3. The feature extreme point should satisfy the ratio threshold $\lambda_{1}/\lambda_{2}>\tau_{\lambda_{12}},\lambda_{2}/\lambda_{3}>\tau_{\lambda_{23}}$.
4. The feature extreme point should satisfy the single-eigenvalue constraints $\lambda_{2}>\tau_{\lambda_{2}},\lambda_{3}>\tau_{\lambda_{3}}$.

Condition 1 can effectively exclude the feature points with holes in the neighborhood or those falling on the edge. Condition 3 can exclude the ambiguity due to symmetry. Condition 4 can ensure that the feature description has the discrimination ability.

#### 2.2.3. Feature Description

In this study, we partially modified the feature descriptor mentioned in ISS [9]. ISS counts the number of neighboring points of the feature point and uses the reciprocal of the number as the weight. This method is time-consuming. We used the distance as the weight. In the actual scanning process, spatial resolution is determined by the distance from the sensor to the object; therefore, in this study, the features were normalized to reduce the effect of the resolutions.

ISS used a discrete spherical grid recursively computed from a base octahedron to divide the spherical angular space into relatively uniformly and homogeneously distributed cells and the radial distances were also evenly divided [9]. The neighborhood points were transformed into the partition space by the LRF, each cell of the ISS descriptor was constructed by counting the weight of the falling point. The weight formula we used is as follows:

(5) $$w=r-\;\|d_{i}\|$$

where r represents the query radius of the feature point neighborhood and $d_{i}$ indicates the distance between the neighborhood point and the feature point. After calculating the cumulative weight in each bin, the feature descriptor was normalized; this normalization reduced the effect of the different resolutions caused by the scanning distances.

#### 2.2.4. Feature De-Duplication

Feature de-duplication helps to reduce the number of features and to speed up query. Firstly, we used the hash map method to reduce de-duplication. The virtual space was divided into voxels, and the global position of the feature point was mapped to a hash bin according to Formula (6). If the hash bin had already been identified, this feature point would be removed as redundancy; if not, this hash bin would be identified. Assuming that the edge length of the divided virtual voxel was u, we mapped the feature point p(x,y,z) in the virtual space as follows:

(6) Hashpos=hash(⌊x/u⌋, ⌊y/u⌋,⌊z/u⌋)

After reducing the feature redundancy, there were still some feature points that were close to each other. The non-maximum suppression method was used to further reduce the number of redundant feature points. In the neighborhood of each feature point, the feature point with the largest $\lambda_{3}$ value was selected as the local feature extreme point, and the other feature points were removed as redundant points.

### 2.3. Feature Database Construction and Query

The construction of the feature query tree directly adopted the LST method proposed in the ISS [9], because this algorithm can quickly organize and query the feature points.

### 2.4. Corresponding Feature Verification

#### 2.4.1. Feature Eigenvalue and Descriptor Verifications

The LST cannot guarantee that the features queried from LST are all correct matches for the current query features, so some methods are needed to filter out the incorrect matching features. As the covariance calculated by the normal of the neighborhood points was used, the sum of the three eigenvalues of the feature point was 1; this made the threshold easier to set. Assume that the descriptor of feature p was $f_{p}$, the eigenvalue of feature point p was $\lambda_{1}^{p},{\;\lambda }_{2}^{p},{\;\lambda }_{3}^{p}$, the corresponding query point was $\left\{p_{i}^{\prime },\ldots ,p_{j}^{\prime }\right\}$, and the descriptor of feature $p_{k}^{\prime }$ was $f_{k}^{\prime }$. The eigenvalues of feature point $p_{k}^{\prime }$ were $\lambda_{1}^{p_{k}^{\prime }}$,$\;\lambda_{2}^{p_{k}^{\prime }}$, $\lambda_{3}^{p_{k}^{\prime }}$. Assume that the eigenvalue threshold was $\nu_{\lambda_{1}}$,$\nu_{\lambda_{2}}$,$\nu_{\lambda_{3}}$. Then, eigenvalues were used to filter out some dissimilar features as follows:

(7) $$\{\begin{array}{c} \lambda_{1}^{p}-\lambda_{1}^{p_{k}^{\prime }}<\nu_{\lambda_{1}}\\ \lambda_{2}^{p}-\lambda_{2}^{p_{k}^{\prime }}<\nu_{\lambda_{2}}\\ \lambda_{3}^{p}-\lambda_{3}^{p_{k}^{\prime }}<\nu_{\lambda_{3}} \end{array}$$

The feature descriptor was normalized, and if two features were the same, the score was 1. We used the following formula to filter out the dissimilar features.

(8) $$\{\begin{array}{c} s\left(p,p_{k}^{\prime }\right)=f*f_{k}^{\prime }\\ s\left(p,p_{k}^{\prime }\right)>\tau_{s} \end{array}$$

where $\tau_{s}$ is the threshold of the similarity descriptor. Empirically, we set $\tau_{s}$ to 0.85.

#### 2.4.2. Pair Distance, Angle, and Direction Verification

There are often many similar features in the actual scene. It is not sufficient to filter out the incorrect matching points by using eigenvalues and descriptions. The distance verification method [20] is a basic verification method. On this basis, we combined the LRF information to further verify the matching pairs. Here, direction and angle filters are proposed to verify the matching pairs. Assuming that $(p_{i},p_{i}^{\prime }$), *i* = 1, 2, …, n, are matching feature pairs, $p_{i}$ is the feature extreme point in current frame coordinate system, $p_{i}^{\prime }$ is the query point from the global (scanning model) coordinate system. The angle verification can be expressed as follows:

(9) $$\{\begin{array}{c} R=R_{LRF}\left(p_{i}\right)R_{LRF}^{T}\left(p_{j}\right){\left(R_{LRF}^{\prime }\left(p_{i}^{\prime }\right)R_{LRF}^{\prime T}\left(p_{j}^{\prime }\right)\right)}^{T}\\ \left(r_{x},\;r_{y},r_{z}\right)=\;\mathrm{degree}\left(R\right)\\ r_{x}<\tau_{r},\;r_{y}<\tau_{p},\;r_{z}<\tau_{y} \end{array}$$

where the degree() function converts the rotation matrix *R* to the rotation angle around the axis. In the ideal case, the rotation matrix *R* is the identity matrix, and $r_{x},\;r_{y},r_{z}$ is 0. Further, $\tau_{r},\tau_{p},{\;\tau }_{y}$ indicates the angle threshold of rotation around the axis. The direction verification can be expressed as follows:

(10) $$\{\begin{array}{c} \mathrm{dir}\left(p_{i},p_{j}\right)=\;R_{LRF}^{T}\left(p_{i}\right)\vec{p_{i}p_{j}}\\ \mathrm{dir}\left(p_{i}^{\prime },p_{j}^{\prime }\right)=R_{LRF}^{T}\left(p_{i}^{\prime }\right)\vec{p_{i}^{\prime }p_{j}^{\prime }}\\ \theta =\;\mathrm{acos}\left(\mathrm{dot}\left(\mathrm{dir}\left(p_{i}^{\prime },p_{j}^{\prime }\right),\mathrm{dir}(p_{i},p_{j}\right)\right))\\ \mathrm{degree}\left(\theta \right)<\tau_{\theta } \end{array}$$

where $\mathrm{dir}\left(p_{i},p_{j}\right)$ means the direction $\vec{p_{i}p_{j}}$ in the local coordinate system of point $p_{i}$, $\vec{p_{i}p_{j}}$ means the direction from point $p_{i}$ to $p_{j}$. Further, $\mathrm{dir}\left(p_{i}^{\prime },p_{j}^{\prime }\right)$ denotes the direction in the local coordinate system of point $p_{i}^{\prime }$, and $\vec{p_{i}^{\prime }p_{j}^{\prime }}$ represents the direction from point $p_{i}^{\prime }$ to $p_{j}^{\prime }$. In the ideal case, the angle between $\mathrm{dir}\left(p_{i}^{\prime },p_{j}^{\prime }\right)$ and $\mathrm{dir}(p_{i},p_{j})$ was 0 and cos(0) was 1; therefore, the $\tau_{\theta }$ was set to a value close to 1.

The number of consistent pairs for each pair was counted, and the pair for this number was less than the threshold was removed.

#### 2.4.3. Feature Clustering

After the distance, angle, and direction verification, we used the dynamic k-means method for pose clustering to obtain the transformation. The specific details of this algorithm are as follows: Assuming that the relative transformation between a series of matching pairs was $\{{\mathrm{pose}}_{i0},\;{\mathrm{pose}}_{i1},\ldots ,$ ${\mathrm{pose}}_{j0}\}$, we selected ${\mathrm{pose}}_{i0}$ as the first cluster center. Assuming that ${\mathrm{pose}}_{i0}$ was the cluster center of gravity of the first group, we calculated the distance between every remaining pose and the pose cluster group centers. If the distance satisfied the threshold, then the pose was added to the current group and was used to update the center position of the current group. If the distance did not satisfy the threshold, then the pose was viewed as a new group. After all poses were added to the groups, the group whose number was more than the threshold was selected as the target group. If the number of points in the target groups was less than the threshold, the relocation failed.

### 2.5. Rigid Transformation Calculation

The use of accurate initial parameters can speed up the registration convergence. Therefore, we used the RANSAC method in each clustered group to obtain a better transformation matrix. In each group, the three matching point pairs were randomly sampled, the transformation matrix was calculated by using the three matching point pairs, and the transformation matrix was used to verify whether the each of the rest of the point pairs was an inner point pair. Finally, the transformation matrix with the largest number of interior point pairs was regarded as the correct transformation matrix. Each group was sorted by the number of inner points from large to small. If there was no significant difference in the number of points in the first group and the second group, we concluded that the interference of similar structures existed, and the relocation was considered to have failed. If the numbers of interior points in the first group and the other group were significantly different, the relocation was considered successful. The transformation matrix was refined by using the ICP algorithm with all the interior points.

In the actual scene applications, firstly, the depth map was downsampled, and the feature points were extracted on the downsampled depth map. Similar features were queried from LST, and then the eigenvalue and the descriptor limitations were applied to filter some incorrect matching point pairs. In the verification stage, the distance, angle, and direction verification method was used to filter out the incorrect matching pairs. Then, the cluster method was used to get the groups whose matching points were more than the threshold; finally, the RANSAC method was used to obtain the final result.

## 3. Experiments and Results

We evaluated the proposed relocation pipeline on the data collected by a small-range visual scanner. All the experiments were implemented in C++ on a PC equipped with a 2.8-GHz Intel i7 CPU and 16-GB memory. We designed offline and online experiments to verify the real-time and relocation rate of the algorithm.

### 3.1. Experimental Object and Parameter Set

**Figure 2:**
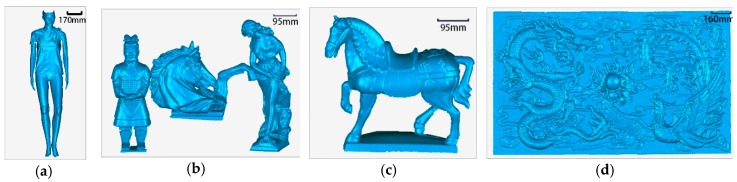
Four experiment objects: (**a**) model, (**b**) small items, (**c**) ceramic horse, and (**d**) emboss.

The four objects were selected from the types of objects that the scanner often scans in actual use. They were used in both the offline experiment and the online experiment, as shown in Figure 2. These four objects had different characteristics. The sizes of “model” and “emboss” were large. The sizes of “small items” and “ceramic horse” were small. The model and ceramic horse objects had smooth surfaces, and the features were not rich. The surfaces of the “small items” and “emboss” objects were rough, and the features were rich on these surfaces.

The range of every frame acquired from the scanner was small, the appropriate scanning distance of the scanner used in this study was between 200 mm and 450 mm, and the range was approximately 260 mm × 170 mm at the distance of 400 mm. In the experiments, the resolution of one frame was 1280 × 1024. The downsampling rate s was set to 4, which implied that the size of the depth map that we used was 320 × 256.

On the basis of experience, some of the parameters were set as follows: the radius of the normal calculation was 3 mm. The radius of the LRF calculation was 6 mm, and the width and the height of the window were N = 20 pixels and M = 20 pixels in the selection of the feature extreme point. The value of $\tau_{area}$ was set between 0.6 and 0.7. $\tau_{\lambda_{12}}$ and $\tau_{\lambda_{23}}$ were set to 1.5. $\tau_{\lambda_{2}}$ was set to 0.01. $\tau_{\lambda_{3}}$ was 0.002. The constraint of the eigenvalues $\nu_{\lambda_{1}}$ was $\lambda_{1}/100$, $\nu_{\lambda_{2}}$ was $\lambda_{2}/5$, $\nu_{\lambda_{3}}$ was $\lambda_{3}/5$,${\;\tau }_{s}$ was 0.85, and $\tau_{r},\tau_{p},\tau_{y}$ were all 10°; $\tau_{\theta }$ was 30°. The maximum 60 features with the largest $\lambda_{3}$ were selected for each frame.

In the pose clustering, thresholds were set relatively broadly; the thresholds classified into one type were 50 mm and 40°, and the distance threshold and the direction threshold in the feature verification stage were 4 mm and 30°. In the RANSAC phase, the difference in the number of the inner points of the first group and the second group used to determine the similar structures was at least one-third the number of inner points in the first group. The minimum number of correct matching features that determined the success of the relocation was 4.

### 3.2. Offline Experiment

An offline experiment was designed to demonstrate the real-time and high relocation correct ratio of the proposed relocation pipeline. During data collection, the scanner normally scans the object, tracks the pose and position, and fuses the data. Therefore, each successfully tracked frame has a rotation ($R_{global}$) and translation matrix ($T_{global}$). The data of the successfully tracked frames and the rotation and translation matrix of these frames were saved. The saved data constituted an acquisition sequence. The frame data were fed into the relocation pipeline in the order of the acquisition sequence. One frame was selected as the relocation frame for every five frames. Firstly, the features were extracted from the feed frame. If the feed frame was not used for the relocation, the feature points were transformed into global feature points by using the transformation matrix ($R_{global}$, $T_{global}$), and these global feature points were inserted into the feature database. If the feed frame was used for the relocation, these feature points were used to query the similar feature points from the feature dataset, and the transformation matrix ($R_{reloc}$, $\;T_{reloc}$) was obtained by using the relocation algorithm. The correctness of the transformation matrix ($R_{reloc}$, $T_{reloc}$) was verified according to formula (11). If the transformation matrix is correct, the frame is considered as a correct relocation frame. The ratio of the number of correct relocation frames to the total number of relocation frames is viewed as the relocation correct ratio.

The specific number of acquisition frames for each dataset, the number of frames selected for testing the relocation, the data size, and the total number of feature points are as shown in Table 1.

**Figure 3:**
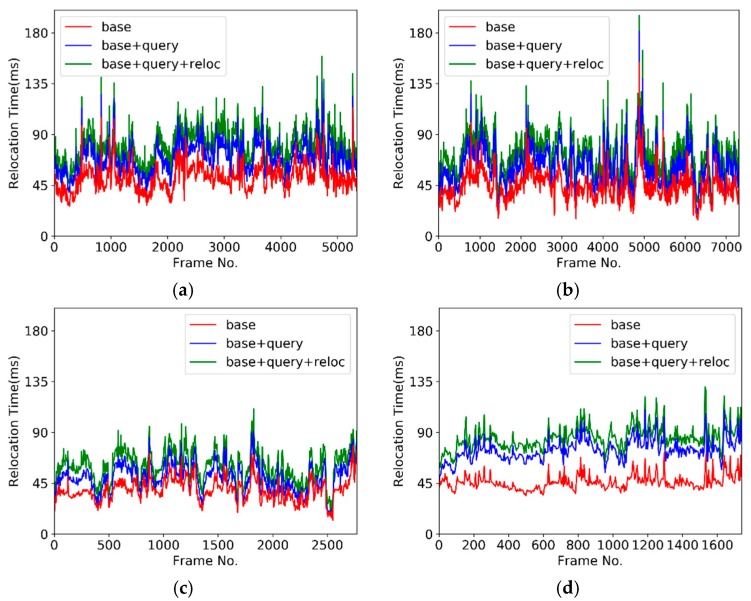
Relocation time cost in the offline experiment: (**a**) relocation time of model, (**b**) relocation of small items, (**c**) relocation time of ceramic horse, and (**d**) relocation time of emboss.

**Figure 4:**
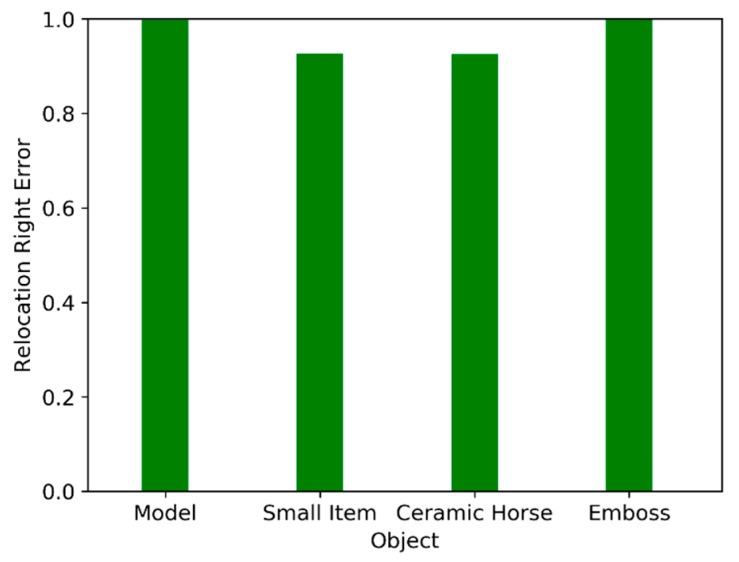
Correct relocation rate of four objects in the offline experiment.

**Figure 5:**
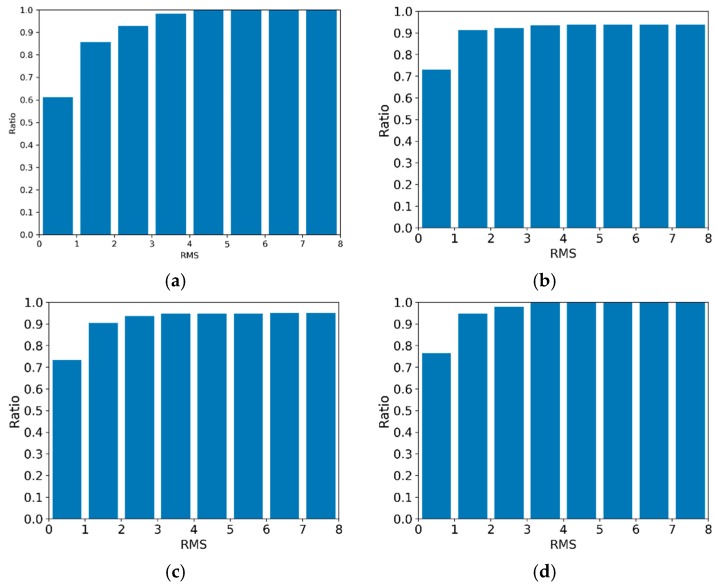
Histogram of cumulative distribution of the relocation RMS errors in the offline experiment. (**a**–**d**) are the RMS error cumulative distribution histogram of the model, small items, ceramic horse, and emboss objects.

**Figure 6:**
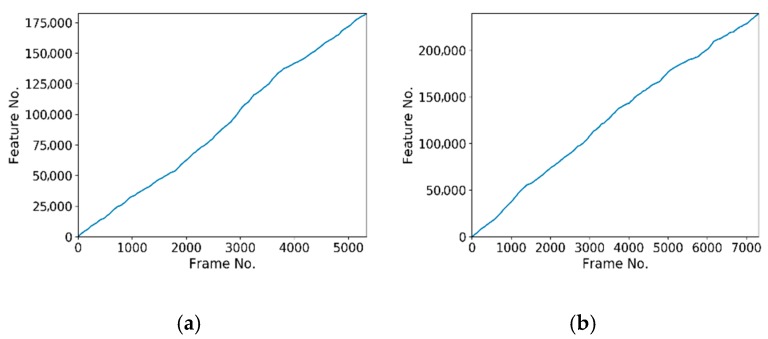

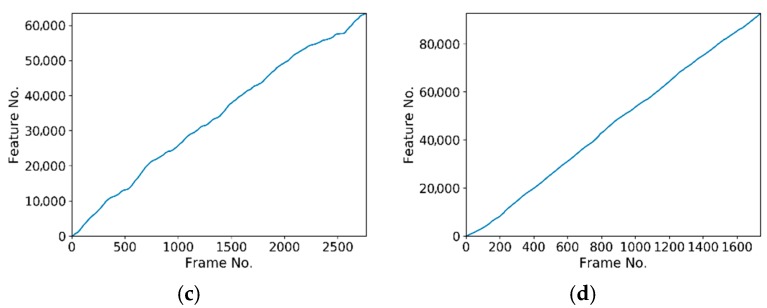
Number of feature points in the offline experiment: (**a**–**d**) are the cumulative number of feature points of the model, small items, ceramic horse, and emboss objects, as the number of frames increases.

The root mean square (RMS) value was used to determine whether the transformation was correct. The specific formula used was as follows:

(11) $$\{\begin{array}{c} \varepsilon_{i}=(R_{reloc}p_{i}+T_{reloc}-\left(R_{global}p_{i}+T_{global}\right)\\ E_{rms}=\;\overset{n}{\underset{i=0}{\sum }}\varepsilon_{i}^{2}/\left(n-1\right) \end{array}$$

where $R_{global}$ and $T_{global}$ represent the precision rotation and translation parameters, which can transform the current frame into a global one; they were obtained by using the GPU-accelerated ICP algorithm in the data scanning process. $R_{reloc}$ and $T_{reloc}$ represent the rotation matrix and the translation matrix obtained by the relocation algorithm. Further, $p_{i}$ denotes the feature points extracted from the current frame, n is the number of feature points, and $\;\varepsilon_{i}$ indicates the distance between the feature points through different transformations. $E_{rms}$ indicates the RMS of the distance. In addition to the RMS statistic, the time cost of the calculation was recorded during the experiment to verify the real-time performance for different numbers of features and frames. In the offline experiment, the time cost of every frame in the relocation, the correct relocation ratio, the relocation RMS error, and the number of feature points were calculated. Figure 3 depicts the relationship between the relocation time and the number of frames. Figure 4 depicts the correct relocation ratio of the four objects. Figure 5 depicts the histogram of the cumulative distribution of the RMS errors. Figure 6 depicts the relationship between the number of feature points and the number of frames.

In Figure 3, for the convenience of identification, the time cost of the depth map downsampling and the feature calculation is called the “base” time, and the time cost of the depth map downsampling and the feature calculation and query is called the “base + query” time. As the computational time of the transformation was small, we combined the time of the feature correlation verification and the time of the transformation calculation, and the time cost of the depth map downsampling, feature calculation, query, feature verification, and transformation calculation is called the “base + query + reloc” time.

### 3.3. Online Experiment

In the offline experiment, one frame out every five frames was selected as the relocation frame, so each test subject had a large number of relocation frames for testing. This facilitated the statistics of time consumption and relocation rate and was beneficial for observing the trend of time consumption and relocation rate as the numbers of frames and features increased. Typically, relocation algorithms are applied to continue scanning for incomplete data or to re-scan in other places; the regions selected for relocation have randomness. Therefore, we chose some regions for the experiment, which were evenly distributed over the surface of the test object. Firstly, we moved the scanner to scan the entire object and then moved the scanner quickly to the appropriate position that maintained a relatively good distance to the selected test range, and at least two thirds of the selected range fell within the field of view of the sensor. The current frame was displayed in the center of the software view; when the relocation was successful, the coarse transformation matrix was obtained, as described in the previous section. This coarse transformation matrix was used to obtain the model frame data by using the raycasting method; the refine transformation matrix was acquired by aligning the current frame to the model frame, and the current frame data were transformed into global data by using this refine transformation. Thus, we determined whether the global position of the current frame was covered with the selected range through naked eye observation. If it was covered, we considered this relocation to be successful. The ratio of the number of correct relocation locations to the number of all selected locations is viewed as relocation correct ratio.

**Figure 7:**
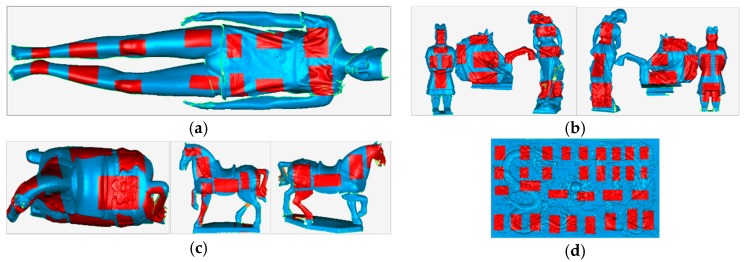
Selected location for the online experiment: the approximate relocation positions are marked by the red rectangles. (**a**–**d**) are selected locations of the model, small items, ceramic horse, and emboss objects.

In the online experiment, we selected the locations evenly to test the performance of the relocation algorithm. As shown in Figure 7, the four objects used for offline testing were used here for the online testing. The selected locations were basically evenly distributed on the surface of the test object.

The information of the experimental data and the experimental results are shown in Table 2. The online experiment was conducted directly after the offline data collection, so the experimental object and the total number of frames were consistent with the offline test. The third column of Table 2 lists the number of selected positions in the four experimental objects. The fourth column lists the statistical average relocation time, and the fifth column lists the statistical relocation rates.

### 3.4. Results

In the offline experiment, the relocation time in the scanning process of the four objects is shown in Figure 3. The “base + query + reloc” curve shows the time shift of the relocation with an increase in the number of frames. The relocation time of the model object was kept below 180 ms when the number of frames was 5345. The relocation time of the small items object was kept below 200 ms when the number of frames was 7320. The relocation time of the ceramic horse object was kept below 135 ms when the number of frames was 2775. The relocation time of the emboss object was below 135 ms when the number of frames was 1746. From Figure 3, we inferred that the relocation time did not increase linearly but fluctuated randomly.

The correct relocation ratio of the four objects is shown in Figure 4. We found that the relocation ratios were all greater than 90%. The relocation RMS error cumulative distribution histogram of the four objects is shown in Figure 5. From Figure 5, it is obvious that the trend of the histogram stabilized when the RMS exceeded 5 mm. During the debugging process, we found that it was appropriate to set the threshold of EMS to 5 mm. This ensured that RT was a good initial value. This initial value made the ICP algorithm converge quickly.

From Figure 6, we inferred that the number of feature points of the four objects reached 182,400, 239,515, 63,400, and 92,674, respectively.

In the online experiment, from Table 2, we inferred that the correct relocation ratio of the model, small items, ceramic horse, and emboss objects, respectively, was 100%, 93%, 100%, and 100%. The average relocation times were 90 ms, 87 ms, 76 ms, and 82 ms, respectively.

### 3.5. Discussion

In the offline experiments, the time consumed by the statistical relocation proved that the proposed relocation algorithm could be used in real time, because each step in the relocation pipeline took less time. Although the number of frames and the number of feature points increased, the relocation time did not increase significantly. This was attributed to the fact that the LST method used a binary tree to organize the feature points. When a feature on the current frame searched for similar features from the database, the feature traversed the LST tree until the leaf node, and on each non-leaf node, the feature traversed the left subtree or right subtree, which determined whether the feature value in the marked dimension was 0. All the features on the leaf node were considered to be similar features. The depth of the tree did not increase very quickly, and the number of judgments was only related to the number of layers, which made the query fast. In the verification step, if there were too many similar features, the verification time was affected, but some simple restrictions, such as limiting the number of similar features, were used to reduce the number of the similar features. Therefore, there was no significant increase in time.

As inferred from Figure 3, we found that both the “base + query” time and the “base + query + reloc” varied with the fluctuations in the base time, and the “query” and “reloc” times were relatively stable. The time of relocation mainly fluctuated with the “base” time. The base time included depth map downsampling, feature calculation, feature extreme point selection, and feature extreme point description. The extracted feature points were generally few, and the time cost of the feature extreme point selection-based grid selection method was small. As the number of feature extreme points per frame was limited to 60, the time cost of the feature extreme point description was extremely small; therefore, the base time was mainly spent on two covariance decompositions (normal calculation and LRF calculation). The reason for this fluctuation was the integrity of the data in the depth map, the occlusion in the data, and other reasons led to the reduction of the invalid data. The less valid the data were, the less the computational time was. The difference in the valid data volume resulted in the fluctuations in the time cost curve.

In the offline test, we observed that the correct relocation rate had reached more than 90%. In theory, if the features on the current frame could search enough correct corresponding features and the verification step removed the wrong matching points, then the correct transformation relationship could be obtained. Further, a correct relocation rate of 90% proved that the constraints of the feature extreme point selection were invalid with respect to ensuring the stability of the feature point position. In addition, one frame out of every five frames was used in the experiment; the ceramic horse and small items objects had more margins in the scanning process than the emboss and model objects (such as the horse legs in the small items object). Therefore, the valid data of the depth map were very small in these tiny places, which led to fewer extracted feature points and led to a relocation failure.

In the online experiment, the relocation time was consistent with the offline test; the reasons for this were analyzed in the offline experiments. The relocation correct ratio was also almost consistent with the offline performance, and the correct relocation rate in the small items object was higher than that in the offline experiment, because the selected regions were not located at the edge or where they could cause occlusions, which made the data relatively complete. These complete data ensured the ability to extract a sufficient number of stable locations. In contrast, the data of one frame at the position of the horse’s leg had a large amount of the invalid data in the small items scan, which caused a relocation failure.

There were also some problems in using this relocation pipeline. In the LST trees, the number of feature points stored on each leaf node was limited. If a feature had too many similar features in the database, these similar features were divided into different leaf nodes. Thus, it was difficult to find the correct matching feature from LST, which would result in a relocation failure. In addition, if many different locations were similar to the current frame, relocation using the current frame failed. Therefore, if there were many regions in the scanning object that were similar to the current frame, the current frame might fail to locate the position and the pose of the scanner.

## 4. Conclusions

We presented a detailed pipeline of a real-time and high-correct-rate relocation algorithm for small-range 3D textureless scanning. The pipeline was divided into four parts, namely feature point calculation, feature database construction and query, corresponding feature verification, and rigid transformation calculation. The experiment proved that the relocation time could be controlled within 200 ms, and the correct relocation rate reached more than 90% in the actual application environment.

The number of feature extreme points and the stability of the feature point position are undoubtedly important factors in determining the correct relocation rate. In this study, the point with the highest curvature in the local region was used as the feature point, and the neighborhood of some of the feature points was disturbed by occlusion or noise. In the future, we intend to use the feature point with the flattest neighborhood to assist with the relocation.

## Figures and Tables

**Table 1:** Objects for offline experiments.

Experimental Object	Total Frame	Relocation Frame	Size (mm × mm × mm)
Model	5345	1069	1700 × 477 × 334
Small Items	7320	1464	555 × 761 × 342
Ceramic Horse	2775	555	416 × 474 × 311
Emboss	1746	349	1222 × 2011 × 443

**Table 2:** Result statistics of online experiments.

Experimental Object	Total Number of Frames	Total Number of Test Locations	Average Relocation Time (ms)	Correct Relocation Rate
Model	5345	14	90	100%
Small Items	7320	28	87	93%
Ceramic Horse	2775	13	76	100%
Emboss	1746	32	82	100%
